# Influence of High Strain Dynamic Loading on HEMA–DMAEMA Hydrogel Storage Modulus and Time Dependence

**DOI:** 10.3390/polym16131797

**Published:** 2024-06-25

**Authors:** Kimberly Cook-Chennault, Sharmad Anaokar, Alejandra M. Medina Vázquez, Mizan Chennault

**Affiliations:** 1 Mechanical and Aerospace Engineering Department, Rutgers University, Piscataway, NJ 08854-5750, USA; 2 Biomedical Engineering Department, Rutgers University, Piscataway, NJ 08554-5750, USA; 3 Department of Chemical Engineering, University of Puerto Rico, Mayagüez, PR 00681-9000, USA; alejandra.medina4@upr.edu; 4 STEM Academy, Stuart Country Day School, Princeton, NJ 08540-1234, USA; mc2807@scarletmail.rutgers.edu

**Keywords:** HEMA–DMAEMA, hydrogel, DMA, storage modulus, loss modulus

## Abstract

Hydrogels have been extensively studied for biomedical applications such as drug delivery, tissue-engineered scaffolds, and biosensors. There is a gap in the literature pertaining to the mechanical properties of hydrogel materials subjected to high-strain dynamic-loading conditions even though empirical data of this type are needed to advance the design of innovative biomedical designs and inform numerical models. For this work, HEMA–DMAEMA hydrogels are fabricated using a photopolymerization approach. Hydrogels are subjected to high-compression oscillatory dynamic mechanical loading at strain rates equal to 50%, 60%, and 70%, and storage and loss moduli are observed over time, e.g., 72 h and 5, 10, and 15 days. As expected, the increased strains resulted in lower storage and loss moduli, which could be attributed to a breakdown in the hydrogel network attributed to several mechanisms, e.g., increased network disruption, chain scission or slippage, and partial plastic deformation. This study helps to advance our understanding of hydrogels subjected to high strain rates to understand their viscoelastic behavior, i.e., strain rate sensitivity, energy dissipation mechanisms, and deformation kinetics, which are needed for the accurate modeling and prediction of hydrogel behavior in real-world applications.

## 1. Introduction

Hydrogels have been an integral part of the medical and engineering fields for over half a century and continue to be an area of intense investigation due to their ideal viscoelastic, hydrophilic, multifunctional, and adaptive properties, where they have been studied for drug delivery [1,2,3,4], environmental remediation via the removal of dyes and heavy metal ions [5], wound healing [6,7], tissue-engineered scaffolds [8,9,10], contact lenses [11,12,13], pH and biosensors [14,15,16], and spinal cord injectables for regeneration [17,18]. Hydroxyethyl methacrylate (HEMA) hydrogels are one of the most extensively studied hydrogels for biomedical and energy storage applications because of their biocompatibility (e.g., non-toxic, non-immunogenic, and tissue compatible) [19,20], stability (e.g., resistance to enzymatic degradation and hydrolysis by acid or alkaline solutions) [21], and physical transparency (facilitating visual monitoring of processes or encapsulated elements for drug delivery, tissue engineering, or biosensors) [22]. HEMA is a monomeric hydrogel that links to other molecules to form a larger polymer chain or three-dimensional network and is water-soluble, soft, flexible, and high in water content. Though these characteristics make it ideal for a variety of applications, it has several drawbacks. 

HEMA hydrogels suffer from mechanical and functional limitations [19]. Thus, modified and co-polymer HEMA-based hydrogels have been developed to enhance HEMA hydrogels’ mechanical properties [23,24,25], electro-responsive properties [26,27,28,29], and physiological responses to environmental conditions (e.g., mechanical loading, ionic concentration [30,31], pH [32,33], and temperature). Many of these HEMA-based hydrogels were developed and studied to understand how certain conditions influence cellular adhesion [34,35], swelling [24,36], porosity [37,38], cellular proliferation [39,40], energy storage [41,42], storage modulus [43,44], and degradation [45,46]. The performance of HEMA-based hydrogels is often a function of the mechanical loading conditions.

Studies focusing on HEMA-based hydrogels’ responses to mechanical loading have proliferated over the last two decades due to recent technological advancements in devices and systems that incorporate hydrogels exposed to new and complex mechanical loading conditions when incorporated into bio-engineered bone and muscle scaffolds, drug delivery (ocular, wound dressings, and transdermal patches), energy storage devices, and soft robotics. Hence, elucidating the mechanical elastic and viscoelastic properties of these materials is vital for designing and optimizing their performance [47,48] in new and emerging applications.

The majority of previous studies focusing on hydrogel mechanical and viscoelastic properties (i.e., elastic modulus, elastic storage, and energy dissipation) have examined hydrogels subjected to static, quasi-static, and low-strain-amplitude conditions. Examples of notable studies on HEMA-based hydrogels subjected to these forms of mechanical loading conditions are provided in Table 1. For example, Jeon et al. [49] found that the swelling behavior, elastic moduli, and degradation rates of photo-crosslinked alginate hydrogels could be controlled by changing the percentage of alginate methacrylation when subjected to static compression loading at a constant strain rate (5%/s) and that the elastic moduli increased with the percentage of methacrylation and decreased with degradation time. Similarly, Piao et al. [50] found that double crosslinking gelatin nanocomposite hydrogels that incorporated graphene oxide exhibited higher compressive mechanical properties (compressive strength, stiffness, and toughness) than neat gelatin hydrogels due to the presence of GO (grafted with glutaraldehyde, GTA). Bektas et al. [51] fabricated interpenetrating network hydrogels comprising Methacrylated gelatin (GelMA) and poly(2-hydroxyethyl methacrylate) (pHEMA) at a ratio of 8:2 and concluded that composite pHEMA–GelMA hydrogels presented a higher compressive modulus (155.49 kPa) than GelMA alone (6.53 kPa). Studies such as these provide the basis for the examination of other HEMA-based hydrogels subjected to mechanical dynamic oscillating loading conditions [52], which are needed to extend and diversify the applications suitable for HEMA-based hydrogels and also elucidate strategies for using inherent material properties to develop tunable devices and systems that meet the individualized needs of consumers and patients.

A few researchers have begun to examine how HEMA-based hydrogels behave when subjected to dynamic oscillating loads. Examples of selected studies of HEMA-based hydrogels subjected to dynamic oscillating mechanical loading conditions (tension, compression, and shear) are detailed in Table 2. This table highlights the methods and measurement tools (DMA and RSA), testing conditions (strain amplitude, strain rate, frequency, and number of samples), and characterization measures (storage modulus, loss modulus, and time) ubiquitously used to quantify viscoelastic material properties of polymer hydrogels. For example, Saunders et al. [53] examined the mechanical storage modulus and mechanical loss of anionic 2-hydroxyethyl methacrylate-co-acrylic acid (HEMA-co-AA) hydrogels as a function of the electrolyte concentration (% wt. potassium hydroxide) and tensile loading frequency at a 1% tensile strain. They concluded that the storage modulus increased as a function of frequency due to the enhanced internal friction of the polymer network, which restricts deformation. Increases in frequency were also found to correspond to increases in the loss modulus due to viscous effects, while frequency effects for both the modulus and loss were more pronounced at lower concentrations of potassium hydroxide until a higher frequency at which the glass transition occurs. Others have examined the storage and loss moduli over a range of frequencies (sweep) to understand the dependence (or independence) of mechanical properties as a function of the load/displacement frequency [54,55]. For example, Lau et al. [55] found that composite resilin-like polypeptide (RLP)–poly(ethylene glycol) (PEG) hydrogel composites subjected to dynamic oscillatory compression over a frequency range of 0.01–10 Hz presented storage moduli values that aligned with the rule of mixtures. In other words, the RLP-PEG micro-structured hydrogels exhibited storage moduli intermediate to the PEG-rich and RLP-rich hydrogels of 22.9 kPa and 6.3 kPa, respectively. Furthermore, all hydrogel storage moduli were independent of frequency within the frequency range evaluated. These prior studies demonstrate that the responses to dynamic oscillating mechanical loading of hydrogels are influenced by the frequency, duration, gel age, strain rate, and direction of the load.

In addition to investigations of various mechanical loading conditions, some scholars have begun to explore hydrogel performance as a function of age. Age testing in hydrogels is needed to understand how crosslinked hydrogels degrade over time and how swelling and shrinkage (which is diffusion-limited) lead to changes in mechanical properties [33,43,56]. For example, Qi et al. [57] demonstrated that mechanical and degradation rates of photo-crosslinking sericin methacryloyl (SerMA) could be tuned by manipulating the methacryloyl (MA) modification degrees to meet various cartilage repair requirements and recorded these differences after 45 days. They concluded that increases in the compressive moduli, stiffness, loss modulus, and storage modulus correlated with increases in MA degrees, while degradation rates were negatively correlated to MA degrees. Additional studies are needed to understand the performance of hydrogels under moderate and high strain-rate conditions over larger frequency ranges and over extended periods of time.

Testing hydrogel mechanical properties at high strain rates helps to illuminate and assess these materials’ ability to withstand and absorb impacts without failure or deformation [58] and ensures that biomedical devices that employ hydrogels can meet safety standards and present reliable performance under dynamic loading conditions [59]. Also, while it is understood that hydrogel mechanical properties can degrade over time, empirical data demonstrative of how this occurs in materials subjected to high strain-rate amplitudes are not readily available. Finally, understanding the behavior of hydrogels subjected to high strain rates is needed to understand the viscoelastic behavior of the hydrogels, i.e., strain rate sensitivity, energy dissipation mechanisms, and deformation kinetics, which are also needed for the accurate modeling and prediction of hydrogel behavior in real-world applications.

**Table 1:** Overview of hydrogels subjected to quasi-static or static compression or tensile mechanical loading conditions.

Material, Testing Method, Strain Rate	Elastic Modulus (kPa)	Strain or Displacement Rate	Time	Reference
MAALG-8 (7.6% methacrylation)	34.3 ± 9.2	Constant compression strain rate @5%/s	24 h	Jeon et al. 2009 [49]
MAALG-14 (13.8% methacrylation)	143.5 ± 4.8		24 h	
		
	174.1 ± 14.9		24 h	
MAALG-25 (25.2% methacrylation), age study	150.0 ± 7.9		7 days	
	110.0 ± 5.5		14 days	
N = 3 for each of the sample sets reported	30.0 ± 1.5		21 days	
Double cross-linked gelatin-GO @0, 0.1, 0.5, 1, 3, 5 mg/mL GO, N = 5 for each sample set reported.				
GH0	23.0 ± 10.0	Constant compression displacement = 1 mm/min	6 days	Piao et al. 2018 [50]
GH1	31.0 ± 12.0			
GH5	31.0 ± 14.0			
GH10	42.0 ± 9.0			
GH30	62.0 ± 18.0			
GH50	58.0 ± 11.0			
SerMA-1 (MA mod. deg. = 0.61 mmol/g) * SerMA-2 (MA mod. deg. = 1.12 mmol/g) SerMA-3 (MA mod. deg. = 1.95 mmol/g) N = 5 for each of the sample sets reported.	4.0 15.0 36.0	Constant compression displacement = 1 mm/min	45 days	Qi et al. 2018 [57]
GelMA GelMA and pHEMA (8:2) N = 3 for each of the sample sets reported.	6.53 155.49	Constant compression displacement = 1 mm/min		Bektas et al. 2020 [51]

* Sericin methacryloyl (SerMA), methacryloyl (MA), and modification degrees (mod. deg.).

**Table 2:** Overview of hydrogels subjected to mechanical dynamic, i.e., Dynamic Mechanical Analysis (DMA) or Rheometric Solid Analyzer (RSA).

Material, Testing Method, Strain Rate	Storage Modulus (MPa)	Loss Modulus (MPa)	Time	Reference
HEMA-DMAEMA	2.23	0.47	72-h	This work, 2024
50% strain amplitude	1.86	0.49	5-days	
DMA—Oscillatory, Compression	1.72	0.47	10-days	
N = 8 for each of the sample sets reported.	2.69	0.86	15-days	
HEMA-DMAEMA	2.43	0.46	72-h	This work, 2024
60% strain amplitude	1.96	0.41	5-days	
DMA—Oscillatory, Compression	1.89	0.38	10-days	
N = 12 for each of the sample sets reported.	2.32	0.53	15-days	
HEMA-DMAEMA	1.51	0.26	72-h	This work, 2024
70% strain amplitude	1.83	0.41	5-days	
DMA—Oscillatory, Compression	1.84	0.38	10-days	
N = 10 for each of the sample sets reported.	2.42	0.62	15-days	
**Material, Testing Method, Strain Rate**	**Storage Modulus** **(KPa)**	**Loss Modulus** **(MPa)**	**Time**	**Reference**
RSA—Oscillatory, Shear, 1% strain amplitude, 0.01–10 Hz, N = 3 samples for each type of hydrogel studied.				
RLP-rich	2.4	57.0	-	Lau et al. 2020 [55]
RLP-PEG_10 **	4.9	28.0	-	
RLP-PEG_25 **	5.0	64.0	-	
RLP-PEG_60 **	5.3	43.0	-	
PEG-rich	2.1	160.0	-	
DMA—Oscillatory, Compression 2.5% strain, 0.05–1 Hz, N = 3 samples for each type of hydrogel studied				
RLP-rich	6.3	62.0	-	Lau et al. 2020 [55]
RLP-PEG_10 **	16.7	70.0	-	
RLP-PEG_25 **	16.2	65.0	-	
RLP-PEG_60 **	18.2	57.0	-	
PEG-rich	22.9	96.0	-	
RSA—Oscillatory, Compression @33% strain amplitude, N = 5 for each of the sample sets reported.				
SMH-1	18.0	14.0	45-days	Qi et al. 2018 [57]
SMH-2	21.0	18.0	45-days	
SMH-3	27.0	20.0	45-days	
DMA—Oscillatory, Tensile 1% strain HEMA-co-AA ***. The number of samples prepared for each sample set was not provided.				Saunders et al. 2012 [53]
2% wt. crosslinker & 0.05 M electrolyte concentration, 0.1 Hz	685.0	8.0	-	
8% wt. crosslinker & 0.5 M electrolyte concentration, 10 Hz	1830.0	430.0	-	

** RLP-PEG gels with micron-scale RLP-rich domains of diameter 10, 25, and 60 mm, respectively. *** 2-hydroxyethyl methacrylate-co-acrylic acid (HEMA-co-AA).

This work examines the elastic and viscoelastic behaviors of HEMA-based hydrogels subjected to high-strain dynamic mechanical compression loading, which has heretofore not been extensively studied. The co-polymer HEMA-2-(dimethylamino) ethyl methacrylate (DMAEMA) is the focus of this study. The monomer DMAEMA was selected because when it is crosslinked with HEMA, it addresses the drawbacks of HEMA hydrogels discussed previously. In particular, DMAEMA introduces ionic character to the hydrogel network, which enhances the mechanical strength and elasticity of the co-polymer [60,61,62]. DMAEMA also contains a tertiary amine group that makes it responsive to pH changes [63,64,65], and depending on the co-polymerization ratio, it can influence the degradation rate of the hydrogel network to provide opportunities for tunable characteristics. Hence, HEMA–DMAEMA hydrogels are the material of focus for this work because they are inert to biological processes, have a water content like living tissues, show resistance to degradation, are permeable to metabolites, are not absorbed by the body, withstand sterilization by heat, and can be fabricated in various shapes and forms. However, additional studies are needed to explore the degradation properties and the sustained and cyclic load-bearing characteristics of these copolymers since these can be limiting factors to the incorporation of these materials into emerging applications [45]. This project seeks to examine the elastic storage modulus, loss modulus, and frequency dependence of stimuli-responsive HEMA–DMAEMA hydrogels as a function of high compression strain rate, time, and frequency.

## 2. Materials and Methods

### 2.1. Materials and Reagents

The monomers—AC334080025, 2-Hydroxyethyl methacrylate (HEMA), stabilized at 97%, and AC215840050, 2-(Dimethylamino)ethyl methacrylate (DMAEMA), stabilized at 99%—were purchased from Acros Organics (Vernon Jills, IL, USA). The crosslinking agent, 335681, Ethylene glycol dimethacrylate (EGDMA), 98%, was purchased from Sigma-Aldrich (St. Louis, MO, USA), and the photo-initiator, 2,2-Dimethoxy-2-phenylactophenone (DMPA), 99%, AC187840000, was purchased from Acros Organics (Vernon Hills, IL, USA).

### 2.2. Sample Preparation

**Figure 1:**
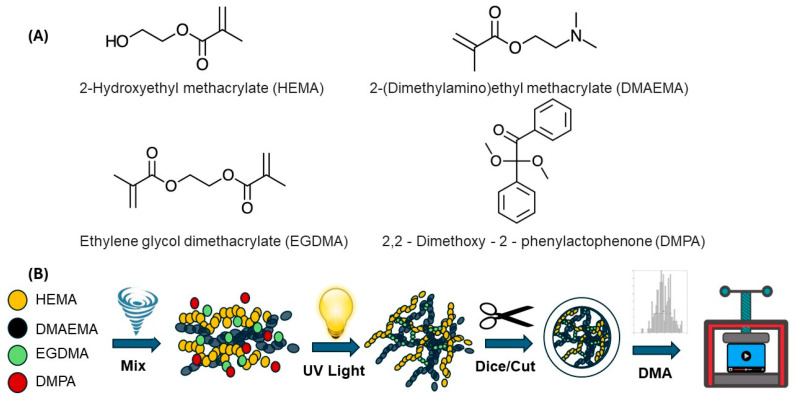
Formulation composition and network formulation for the HEMA–DMAEMA hydrogel. (**A**) Chemical structure of the HEMA and DMAEMA monomers, crosslinker (EGDMA), and photoinitiator (DMPA). (**B**) Schematic illustration of the hydrogel synthesis process, i.e., mixing, UV light at 20 mW/cm^2^, and subsequent cutting of hydrogel to size for mechanical analysis using the DMA.

**Figure 2:**
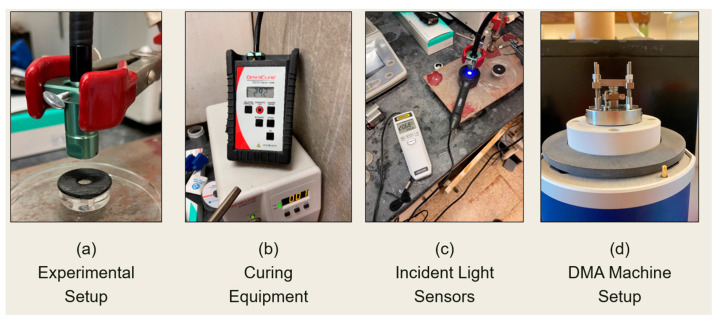
The experimental setup for the UV polymerization and crosslinking process (**a**–**c**) along with the DMA setup (**d**).

A photopolymerization approach (chemical crosslinking), depicted in Figure 1, was used to fabricate the HEMA–DMAEMA hydrogels where ultraviolet (UV) light facilitated the formation of covalent bonds between polymer chains. For this process, the hydrogel was prepared by mixing two monomers—2-hydroxyethyl methacrylate (HEMA) and 2-(dimethylamino) ethyl methacrylate (DMAEMA)—with a crosslinker, namely ethylene glycol dimethacrylate (EGDMA), and a photoinitiator, namely 2,2-dimethoxy-2-phenylactophenone (DMPA), using the ratio of 27.627:5.718:0.467:1 by volume, respectively. The hydrogel mixture was subsequently poured into 1 inch diameter Petri dishes to a height of ~2 mm (according to ASTM D5024-23 [66]). The mixture was then subjected to the ultraviolet light crosslinking procedure detailed in Figure 2.

Prior to applying the ultraviolet (UV) light, a UV mask (30 mm in diameter, with an 8 mm diameter hole, and 2.5 mm thick) was used to cover the Petri dish and focus the spot-curing UV light onto the hydrogels. UV polymerization was performed using an OmniCure S1500 UV Spot Curing System at an intensity of 20 mW/cm^2^ and for a duration of 110 s. An 8 mm guide tip was used at a 25 mm distance from the sample. Incident light was measured at two locations: the guide tip and the sample. The tip level light measurement was performed with the aid of an OmniCure R2000 radiometer. The machine setting on the OmniCure S1500 UV Spot Curing device was set at an incident light intensity of between 100 mW/cm^2^ and 105 mW/cm^2^. For measurements at the sample level, a UAV513AB radiometer was used with its sensing head 25 mm from the end point of the guide tip, and the light intensity was set at 20 mW/cm^2^ and kept stable. The polymerization and crosslinking process was conducted at room temperature.

Cured hydrogel samples were stored in plastic cell culture trays at room temperature in a dark storage cabinet. After 72 h, cured samples were cut to a diameter of 8 mm using a die punch. Samples were subsequently subjected to dynamic oscillatory compression loading in accordance with ASTM standard D5024-23 at four time intervals, i.e., 72 h, 5 days, 10 days, and 15 days from the time of fabrication.

### 2.3. Dynamic Mechanical Analysis

All dynamic mechanical analyses were performed with the aid of a Mettler Toledo dynamic mechanical analysis (DMA) Machine, where a titanium compression clamp was used along with STAR^e^ software (v 16.00). The setup for the DMA compression analyses is depicted in Figure 2d. The samples were subjected to six testing environments (Types 1–6), which are described in Table 3. The storage modulus (E′) and loss modulus (E″) were obtained for each set of samples at four distinct time intervals, i.e., 72 h, 5 days, 10 days, and 15 days from the time of fabrication. The DMA resolutions for force and displacement readings are 1.5 mN and 0.6 nm, respectively. The sensitivity values for the force and displacement sensitivity for the DMA unit are also 1 mN and 5 nm, respectively.

Types 1, 2, and 3 HEMA–DMAEMA hydrogels were subjected to oscillating compression loads at 50% strain amplitudes using a strain rate of 0.25 mm/min. Type 1 and Type 2 sample sets were also subjected to compression loading at a constant frequency equal to 1 Hz; however, Type 1 samples were not stored in a phosphate buffer solution, while Type 2 samples were stored in a 3-pH buffer solution. Type 3 samples were subjected to a frequency sweep from 1–100 Hz but were not stored in a buffer solution. Types 4–6 were subjected to dynamic compression loading at a constant frequency equal to 1 Hz at 50%, 60%, and 70% strain amplitudes, respectively. The strain rate was 0.125 mm/min for Types 4–6 samples.

Age testing was conducted in conjunction with each of the aforementioned test Types to observe the effects of time on the storage modulus and loss modulus of the fabricated samples. Force and displacement amplitude values of the test head were also recorded to shed light on the behavioral characteristics of the HEMA-based hydrogel sample. Tests were conducted at intervals of 72 h, 5 days, 10 days, and 15 days from fabrication. Eight to twelve samples were prepared for each of the test environments described in Table 3. The results reported are shown as averages.

## 3. Results

HEMA–DMAEMA copolymer hydrogels were synthesized and characterized using a dynamic mechanical analysis (DMA) unit. The storage elastic modulus and loss modulus were examined as a function of frequency and time and mechanical properties were captured at time intervals of 72 h and 5, 10, and 15 days. In this study, the Type 1 experimental scenario is the control experiment to which all of the other experimental scenarios (Type 2–Type 6) are compared in Section 3 and Section 4.

**Figure 3:**
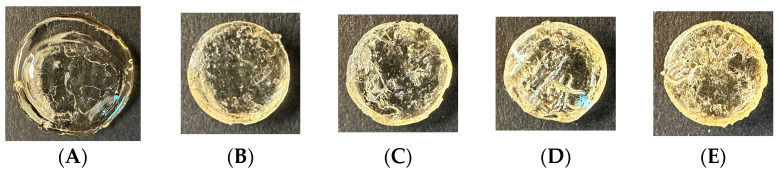
Images of the HEMA–DMAEMA hydrogel (Type 1) captured at time intervals, i.e., (**A**) 0 h, (**B**) 72 h, (**C**) 5 days, (**D**) 10 days, and (**E**) 15 days after synthesis and crosslinking.

Images of hydrogels that were not stored in a buffer solution are provided in Figure 3. As shown in this figure, the gel initially presents a clear amorphous structure where an outer ring that was not subjected to UV is less defined and under-cured. After 72 h, the gels become increasingly cloudy in appearance as a function of time, illustrating reduced transparency and increased light scattering. After 15 days, the hydrogels are almost completely opaque and the overall size of the hydrogel has been reduced. Gels not stored in a buffer solution shrunk ~1% by the 15th day.

**Figure 4:**
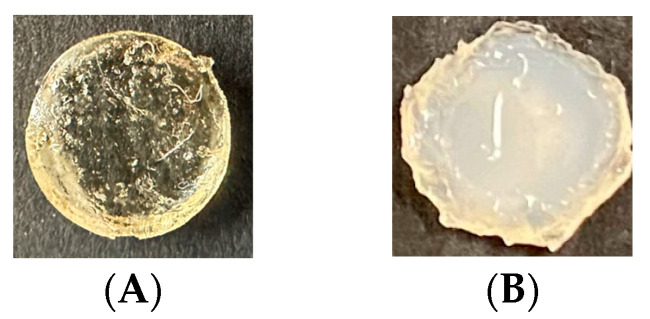
Images of the HEMA–DMAEMA hydrogel captured at 72 h. (**A**) Type 1 gel after removing non-polymerized gel from the periphery and (**B**) Type 2 gel after 72 h of submersion in the buffer solution.

Images of the Type 1 and Type 2 gels, non-buffer and buffered gels captured after 72 h, are provided in Figure 4. These images show a stark contrast to environmental conditions due to the buffer solution, indicating differences in ongoing polymerization and crosslinking that also influence the size and shape of the gels shown.

**Figure 5:**
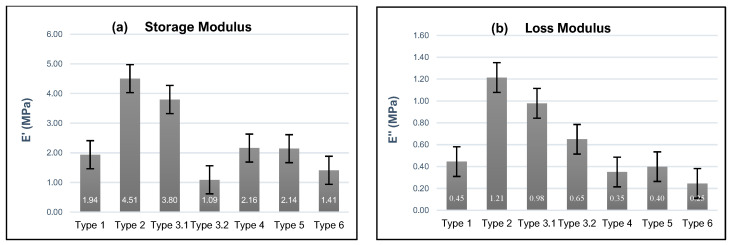
HEMA–DMAEMA hydrogels were subjected to six environments to understand their mechanical behaviors when subjected to high compressive strain rates, where (**a**,**b**) depict the averaged values for the storage and loss moduli, respectively, captured after 72 h.

The storage modulus (E′) and loss modulus (E″) for Types 1–6 are presented in Figure 5a,b, respectively. In these plots, Type 3.1 and Type 3.2 refer to values captured at 1 and 100 Hz, respectively, from the frequency sweep for Type 3 samples. All data presented in Figure 5 were recorded after 72 h. The storage moduli for the non-buffer (Type 1) and 3pH buffer (Type 2) are 1.9 MPa and 4.5 MPa, respectively, indicating that the gel stored in the buffer solution initially has higher stiffness and elasticity observed within the 72 h period. The loss modulus values of Type 1 and Type 2 gels were 0.4 MPa and 1.2 MPa, respectively, illustrating that the gel submersed in the buffer solution has a higher viscous component than the non-buffered gel.

The averaged storage modulus of Type 1 gel (1.9 MPa) is less than the 1 Hz Type 3.1 storage modulus (3.8 MPa) but greater than the 100 Hz Type 3.2 storage modulus (1.1 MPa). In addition, the loss moduli for Type 1 (0.45 MPa) also showed differences compared to Type 3.1 and 3.2, i.e., 0.98 MPa and 0.65 MPa, respectively. The differences in storage moduli and loss moduli between the swept and constant-frequency scenarios demonstrate that the HEMA–DMAEMA viscoelastic properties at 72 h are frequency-dependent.

The responses of Type 1 and Type 4 hydrogels subjected to the same constant 1 Hz frequency and 50% strain amplitudes but different strain rates, i.e., 0.25 mm/min and 0.125 mm/min, are different, where the storage modulus and loss modulus for Type 4 are 2.16 MPa and 0.35 MPa, respectively. The differences in responses demonstrate that the HEMA–DMAEMA gels are strain-rate-sensitive and that the creep, stress relaxation, and viscoelastic flow of the gels may occur differently over the time scale in which the loading occurs.

Type 4, Type 5, and Type 6 gel experimental loading conditions were identical (0.125 mm/min strain rate and 1 Hz frequency) except for the strain amplitudes applied to the samples, which were 50%, 60%, and 70%, respectively. The storage and loss moduli for these testing environments presented an inversely proportional relationship between strain amplitude and storage modulus that could be representative of the nonlinear viscoelastic behavior associated with differences in strain amplitudes.

**Figure 6:**
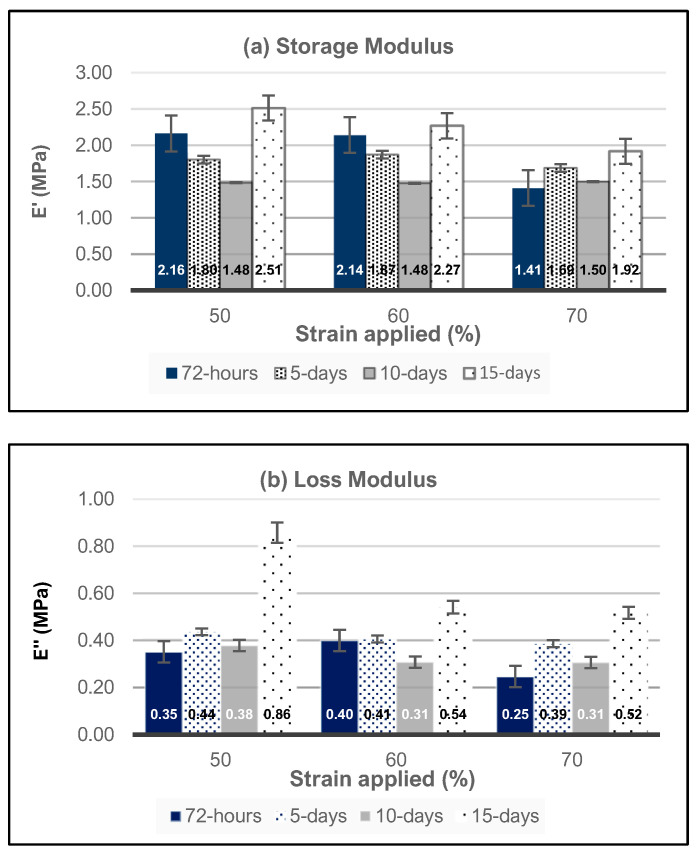
HEMA–DMAEMA hydrogels’ (**a**) storage modulus and (**b**) loss modulus as a function of high strain percentage and time.

The results from the age testing of the samples are shown in Figure 6. To understand the behavior of high-strain-rate compression of hydrogel materials, three strain rates of 50%, 60%, and 70%, i.e., Types 4, 5, and 6, respectively, were explored as a function of time. In general, the storage moduli for all three strain amplitudes decreased from 72 h to 10 days but rebounded up to the highest values by the 15th day, with storage moduli values equal to 2.5 MPa, 2.3 MPa, and 1.92 MPa for strain amplitudes of 50%, 60%, and 70%, respectively. In addition, the storage moduli responses for 50% and 50% are nearly identical when considering the ranges of standard deviation, while those for the 70% amplitude are ~65% of the Type 4 and Type 5 values for the storage modulus. Similar trends are observed for the storage loss for Types 4, 5, and 6 sample sets.

## 4. Discussion

Understanding the mechanical elastic and viscoelastic behavior of hydrogels is important as the former represents the gel’s stiffness or resistance to deformation under static or quasi-static loading conditions, while the latter refers to the gel’s ability to store elastic energy during dynamic oscillatory loading conditions. The loss modulus of hydrogels quantifies the energy dissipated as heat due to viscous effects, i.e., friction and molecular mobility. Since the relationship between the elastic and viscoelastic properties can vary depending on the material composition, structure, crosslinking density, and environmental conditions, this work focuses on the investigation of HEAM–DMAEMA hydrogel viscoelastic behavior observed due to dynamic/oscillatory mechanical loading conditions via analysis of their elastic storage modulus and loss modulus as a function of time, frequency, and buffer solution.

HEMA-based hydrogels are synthetic hydrogels that are typically crosslinked using chemical or physical crosslinking agents. HEMA-based hydrogels are usually amorphous or semi-crystalline and their physical structural form depends on the concentration and type of crosslinking agent. This helps to explain the transformation of the Type 1 hydrogel from 0 h to 15 days depicted in Figure 3. Initially, the gel is well-hydrated and its polymer chains are randomly coiled and loosely intertwined resulting in an amorphous structure that is intact and transparent. However, beyond the 5-day mark, an opaque appearance develops in the gels and increases in density due to the structural changes and alterations in the polymer network, which could result from dehydration, polymer chain aggregation, and microstructural reorganization.

Differences between the buffered (Type 2) gels and the non-buffered gels (Type 1) at 72 h shown in Figure 4 are pronounced compared to the gel’s initial state depicted in Figure 3A. The gel submersed in the 3pH buffer solution for 72 h is less smooth and is cloudy and opaque with significantly less light scattering. In addition, the surface texture is less smooth, and swelling indicative of structural alterations within the network due to the acidic environment is observed.

Since HEMA hydrogels present a positive or negative ionic charge depending on the type of ionic monomer used to crosslink the hydrogel, it is expected that when it is crosslinked with DMAEMA, it will possess an ionic charge due to the DMAEMA component, depending on the storage environment of the samples. The introduction of the KOH (3pH) buffer solution resulted in higher storage modulus values for Type 2 samples (4.51 ± 1.05 MPa) in comparison to Type 1 samples with no buffer solution (1.94 ± 0.24) MPa. These results are similar in range and observation to those found by [43] who observed higher energy dissipation in samples subjected to higher pH buffer solutions. We believe that the higher values of storage modulus in the samples submerged in the buffer solution could be attributed to the increased crosslinking density within the Type 2 gels that result from protonated dimethylamino groups in the DMAEMA that result in positively charged groups. These charges can lead to electrostatic interactions within the hydrogel network that lead to enhanced crosslinking density [67]. In addition, submersion of the gels in the buffer solution exposes them to water molecules that can facilitate interactions between the polymer chains that enhance the network structure, where the increased hydration helps to maintain the integrity of the polymer network that can contribute to a higher storage modulus and improved mechanical stability and stiffness. Also, the protonation of the DMAEMA groups in the low-pH buffer introduces positive charges that lead to electrostatic repulsion between similarly charged groups. The repulsion can lead to an expanded network structure that is more resistant to deformation, which can lead to a high storage modulus. Furthermore, increased chain entanglements and network stiffness can lead to higher energy dissipation during deformation as observed in samples subjected to a buffer solution.

Since the HEMA–DMAEMA hydrogels are viscoelastic, i.e., exhibit both viscous and elastic behavior, in a constant-frequency (Type 1, E′= 1.94 ± 0.24 MPa, E″ = 0.45 ± 0.07 MPa) loading environment, the material has more of an opportunity to relax and dissipate some of the applied stress over time. Hence, the storage modulus for these materials is less than that of the Type 3.1 values at 1 Hz (E′ = 3.80 ± 0.62 MPa, E″ = 0.98 ± 0.13 MPa), which have less time for relaxation compared to the constant-frequency test. This minimized time for relaxation could lead to higher storage and loss. On the other hand, the storage and loss modulus of Type 3.2 (1.09 ± 0.15 MPa @ 100 Hz) are lower than those of Type 1, which may be attributed to the material’s diminishing ability to store elastic energy as the viscous component becomes more dominant, i.e., the network does not have enough time to respond to the changing stress at higher frequencies [68]. Similarly, Type 4 samples, which were exposed to a 50% strain compression load at a low rate of 0.125 mm/min, present higher storage values than Type 1 samples due to the additional time that the samples have to respond to the applied stress, which allows for greater rearrangement of polymer chains within the network and more efficient formation of temporary crosslinks.

The decreased storage and loss moduli for strains of 60% and 70%, sample Types 5 and 6, respectively, in comparison to 50% strain at a strain rate equal to 0.125 mm/min is most likely due to the breakdown of the hydrogel network under increases in loading stress. The breakdown in the hydrogel network may be attributed to several mechanisms, e.g., increased network disruption, chain scission or slippage, and partial plastic deformation. In the case of increased network disruption, crosslinks can function as junctions that hold the polymer chains together, and disruption leads to a reduction in the number of crosslinks, which hinders the material’s ability to store elastic energy. The force applied in compression can also cause breakage (scission) of the polymer chains or lead to slippage of the crosslinks, which results in fluid-like behavior and reduces the gel’s ability to dissipate energy effectively. Also, at exceedingly high strain rates, the hydrogel may also undergo forms of plastic deformation, which means that the deformation is not entirely elastic, and the material may exhibit permanent structural changes, which are irreversible.

The results from the age testing of the samples are shown in Figure 6. To understand the behavior of high-strain-rate compression of hydrogel materials, three strain amplitudes of 50%, 60%, and 70%, i.e., Type 4, 5, and 6, respectively, were explored. The decreases in the storage and loss moduli observed for these sample sets may be attributed to several factors that contribute to the breakdown of the hydrogel network under high strain rates, such as increased network disruptions, chain dynamics, time dependence, and degradation mechanisms. At these high strains, the negative impact of the network disruption usually dominates [69]. The decrease in the modulus from 72 h to 10 days may be primarily related to the viscoelastic behavior of the HEMA–DMAEMA hydrogels, where the initial loading may be recovered over time through the rearrangement of the polymer chains or the reformation of weak interactions. However, the rearrangement process may be incomplete at extremely high strains at 60% and 70%, where some crosslinks may be permanently damaged or broken during the loading. Furthermore, hydrolytic degradation may occur over durations longer than 10 days, which could weaken the polymer chains within the network, further reducing the modulus [70].

## 5. Conclusions

HEMA–DMAEMA hydrogels were fabricated using a photopolymerization approach to examine their viscoelastic properties, e.g., elastic storage modulus and loss modulus as a function of time, frequency, and buffer solution, at high-strain-amplitude mechanical compression loading. As expected, the increased strains resulted in lower storage and loss moduli, which could be attributed to a breakdown in the hydrogel network due to several mechanisms, e.g., increased network disruption, chain scission or slippage, and partial plastic deformation. Furthermore, acute differences in properties were observed between buffered and non-buffered solutions. This study helps to advance our understanding of HEMA–DMAEMA viscoelastic behavior, i.e., strain rate sensitivity, energy dissipation mechanisms, and deformation kinetics, which is needed for the accurate modeling and prediction of hydrogel behavior in real-world applications. Elucidating how HEMA-based hydrogels behave under high-strain-rate dynamic (oscillating) loading conditions will advance the state of the art in how HEMA-based hydrogel polymers can be designed to possess tunable characteristics.

## Figures and Tables

**Table 3:** All samples were subjected to compressive dynamic loading conditions.

Test	Amplitude and Strain Rate	Frequency	Buffer Soln.
Type 1	50% Strain amplitude—0.25 mm/min Strain Rate	1 Hz	No Buffer
Type 2	50% Strain amplitude—0.25 mm/min Strain Rate	1 Hz	3pH Buffer
Type 3	50% strain amplitude—0.25 mm/min Strain Rate	Frequency sweep from 1–100 Hz	No Buffer
Type 4	50% strain amplitude—0.125 mm/min strain rate	1 Hz	No buffer
Type 5	60% strain amplitude—0.125 mm/min	1 Hz	No buffer
Type 6	70% strain amplitude—0.125 mm/min	1 Hz	No buffer

## Data Availability

The original contributions presented in the study are included in the article, further inquiries can be directed to the corresponding author.
